# Antioxidant Effects of Bioactive Glasses (BGs) and Their Significance in Tissue Engineering Strategies

**DOI:** 10.3390/molecules27196642

**Published:** 2022-10-06

**Authors:** Saeid Kargozar, Sara Hooshmand, Seyede Atefe Hosseini, Sara Gorgani, Farzad Kermani, Francesco Baino

**Affiliations:** 1 Tissue Engineering Research Group (TERG), Department of Anatomy and Cell Biology, School of Medicine, Mashhad University of Medical Sciences, Mashhad 9177948564, Iran; 2 Nanotechnology Research and Application Center (SUNUM), Sabanci University, Istanbul 34956, Turkey; 3 Department of Medical Biotechnology and Nanotechnology, Faculty of Medicine, Mashhad University of Medical Sciences, Mashhad 9177948564, Iran; 4 Institute of Materials Physics and Engineering, Applied Science and Technology Department, Politecnico di Torino, Corso Duca degli Abruzzi 24, 10129 Torino, Italy

**Keywords:** bioactive glasses (BGs), oxidative stress, free radicals, tissue engineering, wound healing

## Abstract

Elevated levels of oxidative stress are usually observed following injuries, leading to impaired tissue repair due to oxidation-related chronic inflammation. Several attempts have been made to manage this unfavorable situation, and the use of biomaterials with antioxidant activity is showing great promise in tissue engineering and regenerative medicine approaches. Bioactive glasses (BGs) are a versatile group of inorganic substances that exhibit an outstanding regenerative capacity for both hard and soft damaged tissues. The chemical composition of BGs provides a great opportunity for imparting specific biological activities to them. On this point, BGs may easily become antioxidant substances through simple physicochemical modifications. For example, particular antioxidant elements (mostly cerium (Ce)) can be added to the basic composition of the glasses. On the other hand, grafting natural antioxidant substances (e.g., polyphenols) on the BG surface is feasible for making antioxidant substitutes with promising results in vitro. Mesoporous BGs (MBGs) were demonstrated to have unique merits compared with melt-derived BGs since they make it possible to load antioxidants and deliver them to the desired locations. However, there are actually limited in vivo experimental studies on the capability of modified BGs for scavenging free radicals (e.g., reactive oxygen species (ROS)). Therefore, more research is required to determine the actual potential of BGs in decreasing oxidative stress and subsequently improving tissue repair and regeneration. The present work aims to highlight the potential of different types of BGs in modulating oxidative stress and subsequently improving tissue healing.

## 1. Introduction

A normal tissue healing process includes four overlapping stages of (I) hemostasis, (II) inflammation, (III) proliferation, and (IV) remodeling, which play central roles in the repair process [1]. Numerous experimental studies have emphasized the critical role of immune system cells in advancing tissue repair in vivo. In this regard, activated leukocytes, through releasing reactive oxygen species (ROS) and reactive nitrogen species (RNS), play a central role in tissue repair [2]. However, the excess levels of ROS and RNS are commonly detected following severe tissue injuries, leading to cell damage through distinct mechanisms (e.g., membrane disorganization as well as protein and nucleic acid damage) and subsequently hindering tissue repair [3,4]. Therefore, the balance between ROS/RNS generation and antioxidant defense is crucial for efficient tissue healing in various tissues and organs (e.g., the skin, heart, and bone). From a tissue engineering perspective, specific types of micro/nanosized particles and biomaterials, as well as medicinal herb extracts, have been successfully applied for scavenging the free radicals and preventing excess ROS production [5,6].

Prior studies have presented particular types of nanosized particles as antioxidant substances; ceria (nanoceria), carbon materials (e.g., carbon nanotubes (CNTs)), manganese (Mn), and selenium (Se) are among the most well-studied free radical scavengers [7,8]. Numerous in vitro and in vivo studies have revealed the molecular mechanisms behind these free radical scavengers’ function against oxidative stress. However, the toxicity of nanoparticles has continually been a major concern for biomedical experts and limits their broad administration into the human body. Another group of antioxidant materials includes naturally occurring substances that are being extensively employed for modulating oxidative stress. They are generally classified into enzymatic (e.g., catalase and glutathione peroxidase) and non-enzymatic antioxidants (e.g., flavonoids and polyphenols) groups [9,10]. The latter group is indeed known as active ingredients of medicinal herbs and phytochemicals that are widely utilized for their potent antioxidant activities in tissue engineering and regenerative medicine [11]. The appropriate delivery of natural antioxidants is of utmost importance, and several biocompatible vehicles have been examined for the localized transfer of this kind of antioxidant.

Experimental studies have clarified that applying the above-mentioned antioxidants in combination with other biocompatible materials may result in the generation of tissue replacements with a more potent regenerative capacity [12]. Among the diverse biomaterials used for tissue engineering and regenerative medicine, bioactive glasses (BGs) represent a specific class of inorganic biocompatible materials with the possibility of accelerating both hard and soft tissue healing [13,14,15]. These man-made biomaterials can improve tissue repair and regeneration by enhancing cell growth and proliferation, improving neovascularization, and inhibiting bacterial infection. The chemical structure of BGs provides the possibility of incorporating various metallic and nonmetallic elements into their basic composition, thus generating formulations with extended biological potency. For instance, doping the BG composition with cerium (Ce) leads to the production of antioxidant materials with potent catalase mimetic activity [16]. Moreover, the loading of antioxidant substances (e.g., phytochemicals) to specific types of BGs, e.g., mesoporous BGs (MBGs), was successfully performed for imparting this special activity to the material [17,18,19]. The surface of BGs has also been recognized as a suitable place for grafting bioactive macromolecules [20]; antioxidants (e.g., polyphenols) have been successfully grafted onto BGs for potential use in tissue engineering applications [21].

To the best of our knowledge, this is the first review report specifically discussing the usability and applicability of BGs for modulating oxidative stress and, subsequently, improving wound healing. To this aim, we first introduce free radicals and antioxidants and then deal with the significance of modulating oxidative stress in the tissue healing process. Finally, different types and formulations of BGs will be described as suitable materials for scavenging free radicals and improving tissue repair and regeneration.

## 2. Oxidative Stress and Antioxidants: An Overview

Oxidative stress is described as the imbalance of redox homeostasis due to an irregular increase in free radicals and other reactive molecules, which in healthy conditions play a natural role in cell signaling [22]. In fact, a short-term and relatively small rise in ROS is required for the redox signaling in biological processes such as angiogenesis (HIF-regulated) or inflammation (NADPH oxidases), while a long-term and relatively large increase in ROS induces damage to vital cellular macromolecules, DNA, proteins, or lipids [23,24]. Free radicals (e.g., ROS) or pro-oxidant molecules (compounds that induce oxidative stress) have one or more unpaired electrons that make them extremely reactive for taking electrons from other molecules. They may have a diverse nature depending on the molecules from which they come (oxygen, nitrogen, lipids, etc.). These species are usually produced during cellular metabolism. ROS include free radicals and other powerfully reactive species such as hydroxyl radical (OH^•^), anion radical superoxide (O^2–•^), hydrogen peroxide (H_2_O_2_), peroxyl radical (ROO^•^), and nitric oxide (NO^•^) that are mainly generated by mitochondria [25]. Furthermore, an excess of superoxide free radicals releases free Fe^2+^ from iron-containing molecules, and free iron can form highly reactive radical OH^•^ through the Fenton reaction. Superoxide can react with NO to produce peroxynitrite (ONOO^−^), another highly reactive and toxic free radical (Equations (1)–(6) shown below) [26,27]. Some ROS and RNS, commonly referred to as RONS, can combine together to form other free radicals. Furthermore, an excess of RONS in the mitochondria produces detrimental lipid peroxidation, which increases reactive lipid species (RLS), another source of oxidative stress [7].

Equations (1)–(5):

O_2_ + 1e^−^ + H^+^ ↔HO_2_^•^ ↔ H^+^ + O_2_^−•^ (1)

O_2_^−•^ + H_2_O → OH^−^ + OH^•^ + O_2_ (2)

M^n+^ (metal) + H_2_O_2_ → M^(n+1)^ + OH^–^ + OH^•^ (3)

NOS + L-arginine + O_2_^–•^ + NADPH → NO^•^ + citrulline + NADP^+^ (4)

NOS (Fe (II) heme) + O_2_^−•^ → NOS (Fe(III) heme) + O_2_^−•^ (5)

In normal cells, the presence of uncontrolled oxidative stress triggers death pathways. If the body’s antioxidant defense system fails to neutralize the excess free radicals, the imbalance between the defense system (e.g., antioxidants) and oxidants can cause pathological conditions. On the other hand, inflammatory cells secrete numerous reactive molecules at the inflammation site which, consequently, culminates in worsened oxidative stress. Additionally, a range of reactive species can stimulate an intracellular signaling cascade that has promotive effects on pro-inflammatory gene expression [28]. Thus, oxidative stress and inflammation are closely linked to pathophysiological events and associated with a wide range of chronic diseases, such as diabetic wounds [29]. Furthermore, in tissue engineering, the implanted constructs may face obstacles including exposure to a stressed oxidative environment that can disrupt successful cellular repopulation and tissue regeneration following transplantation. Hence, many strategies have been proposed to tackle these issues; for instance, biocompatible materials with sustainable reactive species scavenging abilities are documented to effectively protect newly formed tissue and engineered constructs from environmental stress. Moreover, safeguarding redox equilibrium is crucial for angiogenesis (an essential step promoting long-term survival and engraftment). On this point, the delivery of antioxidants can preserve the viability of transplants before and after transplantation as well as regulate the oxidative stress in the microenvironment of implanted biomaterials [11,30].

With this in mind, ROS at high concentrations counteract healing processes due to cellular membrane damage. Therefore, it is one of the earliest signals that drive repair as well as regeneration [31], and ROS levels at the site of injury critically affect the regeneration process. However, high levels of ROS can induce severe tissue injuries, even leading to neoplastic transformation [32,33]. The crucial role of reactive species in healing has been shown in systems with NADPH oxidase (Nox) deficiency or antioxidant overexpression.

Antioxidants are substances that can inhibit free radical-mediated oxidative stress and toxic side effects in the human body. The antioxidant defense system controls free radical generation to restore redox homeostasis. In other words, the antioxidant is a stable molecule that donates an electron to unwanted free radical species, neutralizes it and curbs its ability to cause damage. In general, these antioxidants either inhibit or delay cellular damage because of their scavenging properties. These antioxidants have a low molecular weight that allows them to interact with free radicals easily and terminate their chain reaction before damaging vital molecules [34]. Natural cellular antioxidant scavengers include the enzymatic (catalase (CAT), superoxide dismutase (SOD), thioredoxin system (Trx), and glutathione system (GST, GPx, GR)) and non-enzymatic molecules. Non-enzymatic antioxidant substances can be exogenously provided as drugs, although many are naturally acquired via the diet, such as essential fatty acids (omega-3 and omega-6), vitamins (C and E), flavonoids, carotenoids, and trace metals (Se, Mn, Zn) (Table 1). Other antioxidants are endogenously synthesized by cell metabolism, such as coenzyme Q10, melatonin, and reduced glutathione [35].

Nowadays, various antioxidants have been investigated for their therapeutic potential. However, the results of clinical trials have revealed that antioxidants often fail to prevent the progression of ROS-associated diseases, have few benefits, and exert severe side effects at high doses [7]. These unsatisfactory results may stem from low bioavailability, high renal clearance, non-optimal time and duration of therapy, physiological mechanisms that prevent high concentrations in living tissues, and toxicity [36]. In addition, the high concentrations of natural antioxidants may cause toxicity irrespective of the origin. In this regard, large intakes of phenolics were reported to enhance health concerns as to their interactions with proteins; for example, polyphenolic substances can inactivate enzymes [37]. Therefore, it seems necessary to take advantage of biomaterial-assisted approaches in order to target the delivery of antioxidants into the desired locations without the limitations mentioned above.

**Table 1:** A summary of organic and inorganic antioxidant substances that can be used for managing oxidative stress.

Compounds/Examples	Antioxidant Activity	Refs.
**Organic Antioxidants**		
**Carotenoid** **(e.g.,** **crocin, astaxanthin, and β-carotene)**	− Reduction in lipid peroxidation (MDA levels and NO levels) − Increase in the levels of glutathione, antioxidant enzymes (SOD, CAT, and Gpx) and thiol content	[38,39]
**Flavonoid** **(e.g., quercetin and catechin)**	− ROS scavengers and metal ion chelators, − Induction of antioxidant enzymes − Inhibition of pro-oxidant enzymes − Production of the phase II detoxification enzymes − Delaying the onset of lipid peroxidation and preserving the alpha tocopherol level − Preventative activity versus hydrogen peroxide-induced oxyhemoglobin oxidation and loss of heme oxygenase-1	[40,41,42,43,44]
**Phenolic compounds** **(e.g., curcumin and resveratrol, and gallic acid)**	− Scavenging of superoxide anion radicals, hydroxyl radicals, and nitrogen dioxide radicals − Suppressing oxidative stress by modulating Nrf2-HO-1-NF-κB signaling pathways	[45,46,47,48]
**Vitamin C**	− Scavenging of hydroxyl, superoxide radical anion and alkoxyl in biological media as well as reactive nitrogenated species by forming semi-dehydroascorbic acid	[49,50]
**Vitamin D**	− Decrease in the production of ROS − Enhancement in the expression of antioxidant enzymes (CAT, SOD1, SOD2, GPX2, and GPX3)	[51]
**Vitamin E**	− Fight against lipid peroxidation of cell membranes − Ability to mimic CAT, SOD, and oxidase-like activity − Decrease in glutamate-induced intracellular production of ROS or RNS − Reduction of the production of mitochondrial superoxide anion and DNA oxidation by forming a low-reactivity derivative unable to attack lipid substrates	[52,53,54]
**Inorganic Antioxidants**		
**Cerium (Ce)**	− Ability to mimic CAT, SOD, and oxidase-like activity − Decrease glutamate-induced intracellular production of ROS or RNS − Reduction in the production of mitochondrial superoxide anion and DNA oxidation	[55,56]
**Manganese (Mn)**	− It is a part of the antioxidant enzyme superoxide dismutase (SOD) − It has a free radical scavenging capacity	[57,58]
**Selenium (Se)**	− It is incorporated into glutathione peroxidase, an antioxidant enzyme that reduces free-radicals and oxidation in the body − Selenoproteins, as wide range antioxidants, protect the cell from ROS-mediated damages	[59,60]
**Zinc (Zn)**	− Capacity to inhibit lipid peroxidation in liposomes − Zinc deficiency causes increased oxidative stress and, consequently, increased oxidative damage to DNA, proteins, and lipids	[61,62]

## 3. Bioactive Glasses (BGs): A Short Overview

The first BG, trade named as Bioglass, was invented by Hench et al. [63] in 1969 in the USA and originally addressed to bone repair applications. After this discovery, a lot of BG compositions have been reported for various medical applications other than bone healing, such as drug delivery, cancer treatment, and soft tissue engineering [64,65,66]. The original BG composition (45SiO_2_-24.5CaO-24.5Na_2_O–6P_2_O_5_ wt.%, the so-called “45S5”) was based on silica (SiO_2_) as a primary glass network former and had the ability to create bonds with the bone after being implanted in vivo. A sequence of 11 reaction steps describes the bone-bonding processes of silicate BGs to living bone, where the early stages—which may also take place in vitro—yield the formation of a hydroxyapatite layer on the surface of glass [67]. This calcium-phosphate “skin” provides an optimal biological environment for the next reaction stages occurring in vivo, which include cell colonization, proliferation, and differentiation to form new living bone with a good mechanical bond to the implant surface. The thickness of the hydroxyapatite layer has a major impact on bone-bonding ability of the BG as well as on the interfacial shear strength. Generally, an interface thickness of 20 μm offers strong shear strength and interfacial bonding [68]. The porosity, specific surface area and morphology of the BGs control, in general, the formation of new soft or hard tissue. A pore size < 1 μm is responsible for better bioactivity as well as the attachment of cells, while large pores > 50 μm play a major role in tissue formation and vascularization [69,70]. Glass properties can be dictated by the composition/constituents as well as the process parameters and synthesis route (e.g., sol–gel process or melt-quenching method) [71]. BGs can also undergo devitrification through controlled crystallization at an appropriate temperature, thus obtaining bioactive glass ceramics, which typically exhibit better mechanical properties and lower bioactivity as compared to the parent BGs [72].

Several methods have been developed for the synthesis of BGs and their composites, including conventional melt-quenching, sol–gel, flame synthesis, and microwave irradiation. The original 45S5 Bioglass developed by Hench has been prepared by a high-temperature melting process, through the melting of oxides mixed together at more than 1400 °C followed by a quenching step. In the 1990s, soft chemistry strategies emerged, and since then, the sol–gel process has provided a more versatile method to design glasses with very quick bioactive kinetics (apatite formation in a few hours upon contact with biological fluids) [73]. In contrast to the melt-quenching method, sol–gel technology allows the synthesis of BGs of equivalent composition but at a lower temperature. This process is based on the hydrolysis and polycondensation of molecular precursors (alkoxides and salts), which lead to the formation of an inorganic polymeric network at room temperature and ambient pressure. Solvent being trapped within the network explains the gel-like texture; a thermal treatment then allows the removal of solvents and organics as well as the consolidation of the silicate matrix. The other two less-common methods used for BG production are flame synthesis, which consists of baking precursors directly in a flame reactor [74], and the microwave method, that works by dissolving the precursors in water, followed by transfer to an ultrasonic bath and subsequent irradiation [75].

Apart from silicate BGs, B_2_O_3_-based and P_2_O_5_-based BGs have been developed with higher reactivity rates and, hence, higher bioactivity (in the former case) or significant dissolution (in the latter case), which make them also suitable for soft tissue engineering applications [76].

## 4. BGs for Scavenging Free Radicals

**Figure 1:**
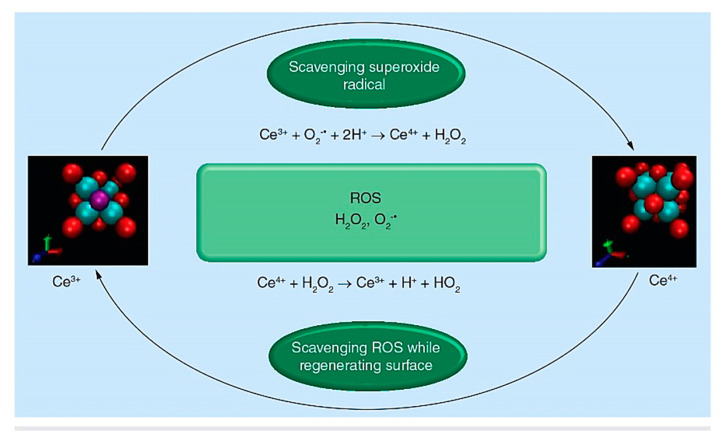
Reactive oxygen species (ROS) scavenging and surface regeneration properties of cerium oxide nanoparticles. Reproduced with permission from Ref. [83].

Up to now, several biomaterials with antioxidant properties have been developed to effectively control ROS levels and modulate the inflammatory response [77]. In this regard, the antioxidant capacity of particular formulations of BGs was evaluated in previously reported experiments with promising results. In fact, BGs with antioxidant activity may be produced by adding specific elements (e.g., Ce) to their chemical composition [78,79,80]. Previously, the effects of the synthesis procedure, composition, and morphology on the catalase mimetic activity of antioxidant BGs were well-investigated by Malavasi and Lusvardi [81]. They added metal oxides (MO, M = Ti, V, Mn, Fe, Co, Cu, Zr, and Ce) to Hench’s 45S5 Bioglass (46.1%SiO_2_–24.4%Na_2_O–26.9%CaO–2.6P_2_O_5_ mol%), Kokubo BG (50%SiO_2_–25%Na_2_O–25%CaO mol%) and MBGs (80%SiO_2_–15%CaO–5%P_2_O_5_ mol%), and then evaluated the potential of these new glasses in inhibiting oxidative stress by testing the hydrogen peroxide (H_2_O_2_) decomposition. Based on their data analysis, the most promising antioxidant properties were confirmed for the Ce-doped BGs [81]. As a matter of fact, Ce is naturally found in dual oxidation modes, i.e., Ce^3+^ and Ce^4+^, and two redox states that lead to the production of cerium dioxide (CeO_2_) and cerium sesquioxide (Ce_2_O_3_). In this regard, it was shown that CeO_2_ nanoparticles could perform free-radical scavenging and oxidative stress attenuation through Ce^3+^/Ce^4+^ redox cycle reactions (Figure 1) [82].

**Figure 2:**
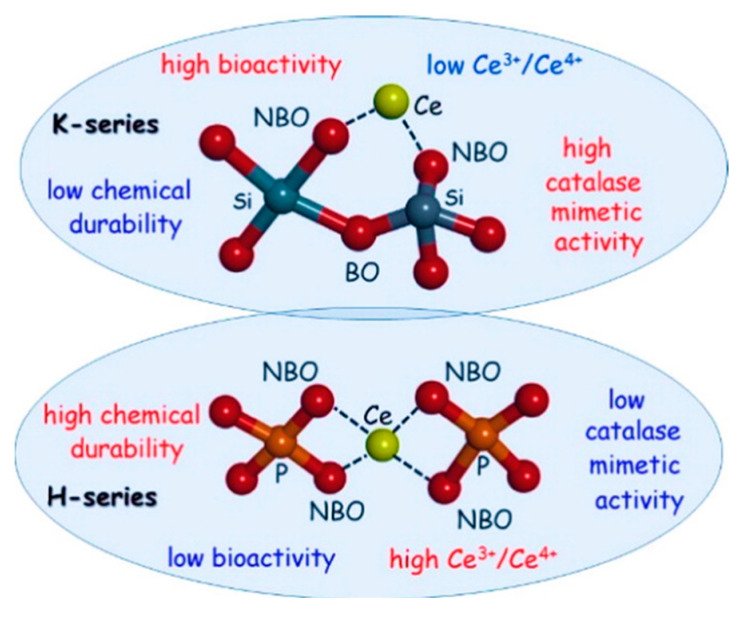
Schematic representation of the decisive role of the chemical composition of BGs, i.e., Hench (H-series) and Kokubo (K-series) glasses, on catalase mimetic activity capacity of cerium (Ce). Reproduced with permission from Ref. [84].

Since Ce is the most common antioxidant dopant that can be added to the glasses, its catalase mimetic activity was studied in two different BG formulations, i.e., Hench’s (46.2%SiO_2_–24.3%Na_2_O–26.9%CaO–2.6P_2_O_5_ mol%) (H-series) and Kokubo’s (50.0%SiO_2_–25.0%Na_2_O–25.0%CaO) (K-series) glasses [84]. The reported results have clarified the critical role of the chemical composition on the catalase mimetic activity of Ce. Indeed, the presence of phosphate groups in the intimate glasses’ structure and/or in the environment (simulated body fluid (SBF) solution vs. pure water) lowered their catalase mimetic activity because phosphate groups stabilize the Ce^3+^ species to form the CePO_4_ insoluble phase, which inhibits the interconversion process between Ce^3+^ and Ce^4+^ (Figure 2). The low cytotoxicity and broad spectrum of the bacteriostatic activity of such glasses have also been verified by classical molecular dynamics simulations [85]. The negative role of phosphate units in the glass network on catalase mimetic activity of Ce-doped BGs has also been reported elsewhere [86,87].

It should be highlighted that Ce at relatively high concentrations (above 5.3 mol%) was able to extremely reduce the bioactivity of BGs because of the formation of insoluble Ce-containing phases, such as CePO_4_: in fact, the cerium ions released by the glass surface react quickly with the phosphate ions of the SBF forming the CePO_4_ insoluble phase and making phosphate ions unavailable for hydroxyapatite formation [87]. which is key to allow bone bonding and regeneration in vivo. Therefore, it is crucial to carefully select the dosage of Ce in the glass composition in order to maintain both therapeutic effects, i.e., ROS scavenging and bioactivity.

**Figure 3:**
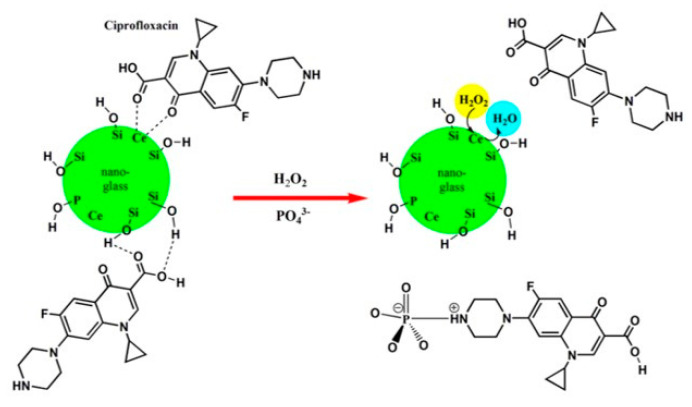
Schematic representation of Ce-doped nano-bioactive glasses based on 60SiO_2_-(10-x)B_2_O_3_-25CaO-5P_2_O_5_-xCeO_2_, in mole% (x = 0 and 5 mol%) as multifunctional bone fillings for drug delivery of ciprofloxacin. Reproduced with permission from Ref. [94].

One possible solution for utilizing a high amount of Ce in the glass network relies on the doping of sol–gel-derived BGs instead of the melt-derived counterparts, as the former class of BGs is more bioactive as compared to the latter due to the inherent nanoporous texture and larger specific surface area [71,88]. Emphasizing the particle size significance, CeO_2_-incorporated nanosized BGs showed a faster apatite formation, enhanced dissolution rate, and higher protein adsorption as compared with their bulk counterparts [89,90]. In order to study the atomic-scale properties of nanosized glasses, molecular dynamics (MD) simulation has been applied as a powerful common tool, supporting the interpretation of experimental trends [78,91]. Using classical core-shell MD simulations, Pedone et al. evaluated the antioxidant activity of two nano-BG compositions, i.e., Hench’s Bioglass (46.1SiO_2_–24.4Na_2_O–26.9CaO–2.6P_2_O_5_ mol%) and Kokubo’s phosphate (P)-free soda lime silicate glass (25Na_2_O–25CaO–50SiO_2_ mol%) doped with 3.6 mol% of CeO_2_ [92]. The authors found that the different catalase mimetic activity of the two BGs was due to the Ce^3+^/Ce^4+^ ratio exposed at their surface (3.5 and 1.0 in bulk and 13 and 2.1 at the surface of the Hench’s and Kokubo’s glasses, respectively). Moreover, a very high Ce^3+^/Ce^4+^ ratio caused a reduction in antioxidant properties due to the necessity of both oxidation states of Ce for the dismutation reaction catalysis of hydrogen peroxides. The active cerium sites within 45S5-based BGs have been accurately described in a study by Benedetti et al. in order to understand the functionalities at the atomic scale by investigating the local structure around Ce ions. The presence of small amounts of Ce within the BG matrix may render an antioxidant property for bone tissue regeneration. A contracted Ce-O first shell distance (2–3%) concerning bulk oxides was identified in complete agreement with the results of MD simulations [93]. Ce-doped nano-BGs with a composition of 60SiO_2_–(10-x)B_2_O_3_–25CaO–5P_2_O_5_–5CeO_2_, (mol%) was also proposed as a multifunctional bone filler with the ability to deliver drugs (ciprofloxacin) (Figure 3) [94]. This glass was demonstrated to decrease reactive oxygen activity through its excellent catalytic activity as a result of the fast interchange of the oxidation states of Ce^3+^/Ce^4+^. The authors concluded that the drug release behavior is determined by both the glass composition and the oxidative stress condition.

The use of BG-polymer composites may provide additional advantages for tissue engineering and drug delivery applications. For example, polymers may act as a cross-linking bridge to enhance the interfacial interaction between glasses and loaded drug, which can significantly increase the drug-loading capacity while avoiding initial burst release [95]. In order to take benefit from BGs and polymers simultaneously, Dziadek et al. prepared polycaprolactone (PCL)-BG composites as carriers for antioxidant polyphenols (PPh) extracted from Salvia officinalis L. [96]. The existence of PPh in the composite films enhanced their mechanical properties and provided antioxidant activity. The authors have shown that PPh release kinetics can be modulated by the use of the sol–gel-derived BG particles (40SiO_2_–54CaO–6P_2_O_5_ mol%). The films containing the lowest concentration of PPh (1.5 w/w) exhibited good cytocompatibility, significantly increased alkaline phosphatase (ALP) activity, and induced the expression of bone extracellular matrix proteins (osteocalcin and osteopontin) in human normal osteoblasts in vitro; in contrast, they reduced the production of intracellular ROS in macrophages. Furthermore, the composites loaded with PPh showed antibiofilm properties against Gram-positive and Gram-negative bacteria. The results indicate that the developed constructs represent potential multifunctional biomaterials with a wide range of tunable physicochemical and biological properties that are beneficial for tissue engineering. In another study, ferulic acid, which is known to be an antioxidant phenolic phytochemical, was utilized for developing chitosan–BG–ferulic acid (CS-BG-FA) composite coatings by using the alternating current electrophoretic deposition (AC-EPD) technique [97]. The prepared construct was compatible with MG-63 human osteoblast-like cells and showed an effective bactericidal activity against both Gram-positive and Gram−negative bacteria. However, no specific assay was carried out by the authors for determining the antioxidant performance of the composites.

## 5. Mesoporous Bioactive Glasses (MBGs) as Platforms for the Delivery of Antioxidants

As earlier mentioned, MBGs are an extraordinary class of BGs that hold great promise in drug delivery and tissue engineering strategies [98,99]. Up to now, a huge number of MBGs have been developed and applied for rendering particular biological activities. For instance, MBGs with a high surface area to volume ratio exhibit superior bioactive behavior and better in vivo osteogenesis as compared to conventional glasses [100,101]. Additionally, MBGs can be loaded with different types of biomolecules (e.g., growth factors) and provide a drug delivery system for accelerating tissue repair and regeneration [102].

Most antioxidant MBGs have been prepared by incorporating Ce into their chemical composition [103]. Since the incorporation of Ce into melt-derived BGs leads to a drastic decrease in their bioactivity, sol–gel-derived MBGs were used for preparing Ce-containing antioxidant glasses in various experiments. In this regard, 80SiO_2_–15CaO–5P_2_O_5_ and 80SiO_2_–20CaO (mol.%) MBGs samples doped with 5.3 mol.% of CeO_2_ showed good catalase activity while still exhibiting proper bioactivity properties [104]. In 2021, El-Fiqi et al. evaluated the effect of Ce doping on the structural, physicochemical, catalase-mimic, and biological properties of MBGs. The composition was a binary 85% SiO_2_–15% CaO glass in which Ce (0, 5, and 10 wt%) partially replaced CaO [105]. These ultrasmall-sized MBGs (<30 nm) were successfully developed by the ultrasound-assisted sol–gel method. The presence of Ce^3+^ and Ce^4+^ (72.57 and 27.43%, respectively) was detected at the MBG surface. The glasses showed a high antioxidant effect (catalase-mimic activity) without causing any adverse impacts on bioactivity and cytocompatibility. In order to promote the therapeutic effects of MBG nanoparticles (MBGNs), their endowment with additional antioxidant properties has become of great interest to control the oxidative stress associated with bone remodeling defects. As a result, the pro-osteogenic and anti-inflammatory activities of antioxidant mesoporous spherical Ce-doped MBGNs (100–300 nm) have been verified by Zheng et al. using a two-step strategy [106]. First, a microemulsion-assisted sol–gel method was applied to synthesize MBGNs (70SiO_2_–30CaO, mol%), and then a post-impregnation approach was developed for the modification of MBGNs with Ce. With a concentration of Ce^4+^ (relative molar percentage of 74%) higher than that of Ce^3+^, the obtained Ce-doped MBGNs at 1 mg/mL showed no cytotoxicity against fibroblasts. Moreover, a reduction in the expression of genes responsible for oxidative stress in macrophages (J774a.1) was observed after incorporating Ce into MBGNs. Ce-doped MBGNs also suppressed pro-osteoclastogenic responses due to their pro-osteogenic activities, which make them promising candidates as advanced biomedical devices for targeting infected bone defects and inflammatory bone diseases (e.g., osteoporosis) [106,107].

In addition, to incorporate antioxidant elements, MBGs can be loaded with antioxidant natural or synthetic macromolecules for modulating oxidative stress and accelerating tissue healing. On this matter, gallic acid, polyphenols (POLY), and anthocyanins were successfully loaded into Ce-doped MBGs to enhance their antioxidant activity [17]. The results clarified that unloaded Ce-MBGs have only a marginal capability of SOD-like activity, while the samples loaded with the biomolecules, especially POLY, revealed a substantial improvement in the SOD-like activity.

Ce-containing MBG-derived 3D scaffolds were also successfully developed with drug delivery ability for possible use in tissue engineering applications [108]. These scaffolds were fabricated by using poly(methyl methacrylate) (PMMA) as a sacrificial template, and the presence of Ce was confirmed in both oxidation states Ce^4+^/Ce^3+^, in the scaffolds. In some studies, Ce-doped MBGs were embedded into polymeric matrices to make composites with antioxidant activity and high tissue regeneration capacity. For instance, Ce^3+^/Ce^4+^-containing MBGs were previously added to alginate beads for bone tissue engineering applications [109]; the results showed the beads having 1.2 and 3.6 CeO_2_ mol% could counteract the oxidative stress without a negative impact on the proliferation of pre-osteoblastic cells MC3T3-C1 cells. However, cell differentiation was decreased as a function of Ce-content in the samples.

Shruti et al. [110] also incorporated curcumin in Ce-, Ga- and Zn-doped MBGs (basic composition 80SiO_2_–15CaO–5P_2_O_5_ mol.%) to obtain a triply functional biomaterial combining an apatite-forming ability (which is key in the context of bone regeneration) and ion/drug release. The release profiles of curcumin from these glasses were able to exert pharmacological activities, thus showing great promise in overcoming the typical limitations of curcumin (e.g., insolubility in water, poor bioavailability); however, the antioxidant properties in vitro/in vivo were not specifically investigated in that study. 

## 6. Conclusions and Future Outlook

Controlling oxidation could be the key that opens the doors of a new type of targeted biomedicine with huge impact and opportunities for improving therapy. This is a partially unexplored land requiring, firstly, a better knowledge of the biomolecular mechanisms behind oxidation and related physiological/pathological effects, as well as the relationship between oxidation and immunomodulatory pathways. Implantable BGs can play a major role in this scenario as they are able to indeed exert antioxidant effects on cells and tissues through two main modalities, i.e., the release of antioxidant ions or antioxidant biomolecules. In this regard, great promise has been shown by MBGs as they can act as carriers for the uptake and delivery of both therapeutic agents, alone or simultaneously. In fact, metallic elements such as cerium can be incorporated into the glass network during the synthesis process and then released upon contact with biological fluids according to controllable kinetics. On the other hand, cerium was reported to delay the apatite-forming ability of BGs, and hence the bioactivity, due to the formation of competitive and insoluble cerium phosphate phases. In order to manufacture BGs with higher bioactivity, a suitable morphology should be selected. In this regard, MBGs with high specific surface area due to nanotexturing and nanosize allow the bioactivity-related concerns of Ce-containing glasses to be overcome. Furthermore, a range of organic molecules, even characterized by challenging properties such as insolubility in water, could be loaded into the nanosized pores of MBGs, from which they can then exert the desired therapeutic action. This is the case of curcumin, which can be potentially used for the treatment of various oxidation-related diseases ranging from skin wounds to cancer. How to govern the concurrent release of ions and biomolecules in terms of synergistic/antagonistic interactions is one of the great challenges for BG researchers and biomaterials scientists in general.

Moreover, the surface reactivity of BGs can be effectively exploited for grafting procedures and functionalization strategies, involving both the outer surface of glass products and the walls of internal nanopores (as in the case of MBGs), thus further expanding the versatility of such biomaterials.
